# Increasing proportion of carbapenemase-producing *Enterobacteriaceae* and emergence of a MCR-1 producer through a multicentric study among hospital-based and private laboratories in Belgium from September to November 2015

**DOI:** 10.2807/1560-7917.ES.2017.22.19.30530

**Published:** 2017-05-11

**Authors:** Te Din Huang, Pierre Bogaerts, Catherine Berhin, Martin Hoebeke, Caroline Bauraing, Youri Glupczynski, on behalf of a multicentre study group

**Affiliations:** 1 National Reference Laboratory for Antibiotic-Resistant in Gram-negative bacilli, CHU UCL Namur, Université Catholique de Louvain (UCL), Belgium; 2 The members of the group are listed at the end of the article

**Keywords:** Epidemiology, carbapenem resistance, community, colistin resistance

## Abstract

Carbapenemase-producing *Enterobacteriaceae* (CPE) strains have been increasingly reported in Belgium. We aimed to determine the proportion of CPE among *Enterobacteriaceae* isolated from hospitalised patients and community outpatients in Belgium in 2015. For the hospitalised patients, the results were compared to a previous similar survey performed in the same hospitals in 2012. Twenty-four hospital-based and 10 private laboratories collected prospectively 200 non-duplicated *Enterobacteriaceae* isolates from clinical specimens. All isolates were screened locally by carbapenem disk diffusion using European Committee on Antimicrobial Susceptibility Testing methodology. Putative CPE strains with inhibition zone diameters below the screening breakpoints were referred centrally for confirmation of carbapenemase production. From September to November 2015, we found a proportion of clinical CPE of 0.55% (26/4,705) and of 0.60% (12/1,991) among hospitalised patients and among ambulatory outpatients respectively. *Klebsiella pneumoniae* (26/38) and OXA-48-like carbapenemase (28/38) were the predominant species and enzyme among CPE. One OXA-48-producing *Escherichia coli* isolated from a hospital was found carrying plasmid-mediated MCR-1 colistin resistance. Compared with the 2012 survey, we found a significant increased proportion of clinical CPE (0.55% in 2015 vs 0.25% in 2012; p = 0.02) and an increased proportion of hospitals (13/24 in 2015 vs 8/24 in 2012) with at least one CPE detected. The study results confirmed the concerning spread of CPE including a colistin-resistant MCR-1 producer in hospitals and the establishment of CPE in the community in Belgium.

## Introduction

Acquired carbapenemases in *Enterobacteriaceae* have been reported extensively worldwide [1]. Asymptomatic carriage and infection caused by carbapenemase-producing *Enterobacteriaceae* (CPE) isolates currently raise major public health concern for individual therapeutic management and for collective infection control [2]. The prevalence of carbapenem resistance and the types of carbapenemases found in Europe vary between countries [3,4] and since 2010 an increase in the isolation rate of CPE from various European countries has been reported [5]. In Belgium, the last multicentric survey performed among 24 hospitals in 2012 [6] showed an overall proportion of 3.5% of carbapenem non-susceptible *Enterobacteriaceae* (CNSE) and an estimated proportion of 0.28% of CPE among *Enterobacteriaceae* isolates using Clinical and Laboratory Standards Institute (CLSI) carbapenems susceptibility breakpoints as screening criteria [7]. Further, one third (8/24) of the participating hospital laboratories had isolated one or several CPE isolates in their institution during the study period [6]. The national surveillance programme of CPE established in January 2012 by the Scientific Public Health Institute together with the National Reference Centre showed a yearly increase from 2012 to 2014 of number and proportion of hospital-based and of private laboratories reporting confirmed CPE [8].

The present cross-sectional survey aimed to determine the evolution of the proportion of CNSE and of CPE among *Enterobacteriaceae* isolates collected in Belgian hospitals and to gain insight of their epidemiology outside hospitals in specimens collected from community outpatients sent to private laboratories.

## Methods

### Study design, inclusion criteria and testing at participating centres

In 2015, 24 hospital-based laboratories that participated to the survey in 2012 serving around 25% of all acute hospitals in Belgium (hospital size ranging from 288 to 1,803 beds; median number of 747 beds per hospital) were requested to collect consecutively 200 *Enterobacteriaceae* isolates cultured from clinical samples of hospitalised patients (screening samples such as stools or rectal swabs were excluded) over a period of 2 months (i.e. from September to November). Only the first isolate of the same species per patient was included. The sample collection, culture and bacterial identification were performed using local procedures. In addition, 10 private laboratories (representing around 20% of all private laboratories in Belgium) serving general practitioners participated as well using the same study protocol and were asked to test 200 *Enterobacteriaceae* isolates cultured from clinical samples of outpatients in the community.

All isolates were tested locally for susceptibility to meropenem (10-µg) and ertapenem (10-µg) by disk diffusion method according to European Committee on Antimicrobial Susceptibility Testing (EUCAST) guidelines [9] and all inhibition zone diameters were recorded. All testing materials purchased from the same manufacturer (BioRad, Marnes-la-Coquette, France) had the same manufacture batch number in order to avoid inter-lot variations. *Escherichia coli* ATCC 25922 was tested as quality control strain in each centre during the survey.

*Enterobacteriaceae* isolates showing a decreased inhibition zone diameters according to EUCAST screening breakpoints [10] to any of the two carbapenems disks (ertapenem < 25 mm and/or meropenem < 25 mm) were defined as putative CPE (PCPE) and had to be referred to the reference laboratory.

### Characterisation of resistance mechanisms and data analysis

All PCPE isolates sent to the reference laboratory were tested for carbapenemase production by the electrochemical imipenem-hydrolysis based BYG Carba test [11] and underwent multiplex PCR targeting *bla*_VIM_, *bla*_IMP_, *bla*_NDM_, *bla*_KPC_ and *bla*_OXA-48_ for the detection of carbapenemase encoding genes [12]. The PCR-positive amplicons were sequenced using an external service company (Macrogen Inc., Seoul, South Korea). The sequence obtained was compared with the genes present in GenBank and aligned with the reference gene cited at the Lahey Clinic [13]. 

For confirmed CPE isolates, minimal inhibitory concentration (MIC) values of antimicrobial agents including carbapenems were determined by broth microdilution method (Sensititre, Trek Diagnostic Systems, Cleveland, US). The MIC value of temocillin was determined by Etest method (bioMérieux, Marcy-l’Etoile, France). Susceptibility categorisation was interpreted according to EUCAST interpretative criteria [9] for all antimicrobial agents except temocillin (breakpoints according to Fuchs et al.) [14]. Colistin-resistant isolates were further tested by end-point PCR for the presence of the plasmid-mediated colistin resistance *mcr-1* gene [15].

The proportion of CNSE as defined by the EUCAST disk diffusion susceptibility breakpoints (ertapenem <25 mm and/or meropenem <22 mm) and of CPE isolates were calculated based on inhibition zone diameters of all isolates provided overall in hospitals or in private laboratories, for each centre and also by species or genus. Following the application of harmonised microbiological criteria (the ertapenem and meropenem discs zone diameters recorded in 2012 were reinterpreted using EUCAST interpretative criteria), results from 2015 were compared with those in 2012 for hospital-based laboratories.

Clonal relatedness of the carbapenemase-producing *Klebsiella pneumoniae* isolates was investigated by the DiversiLab system (bioMérieux, Marcy l’Étoile, France) using the repetitive extragenic palindromic (rep)-PCR methodology [16]. Isolates with > 95% similarity were considered related.

## Results

In total, 4,705 and 1,991 non-duplicate *Enterobacteriaceae* isolates were screened by the 24 hospital-based and by 10 private laboratories respectively from September to November 2015. A median number of 200 isolates per laboratory was achieved and all but one centre reached > 195 isolates screened. The distributions of origins of screened isolates were highly comparable to 2012 with urine (2,642/4,705; 56%) and medical wards (1,818/4,705; 39%) representing the principal origins of the isolates screened and the main sources of CNSE isolates in hospitals (data not shown). The highest proportion of CNSE isolates in hospitals was found in respiratory tract (36/714; 5.0%) and in the intensive care units (30/711; 4.2%). On the other hand, urinary origin (1,902/1,991; 96%) of screened isolates was as expected largely predominant in private laboratories.

The number and the proportions of CNSE according to EUCAST susceptibility criteria and of CPE among tested isolates per species are reported and compared with the 2012 study results in Table 1.

**Table 1:** Species/group distribution and proportion of CNSE (according to EUCAST interpretative criteria) and of CPE among isolates screened by 24 hospital-based and 10 private community-serving laboratories in Belgium, 2012 (n = 4,471) and 2015 (n = 6,696)

Species or group	2015 survey					2012 survey				
	Screened	CNSE	%CNSE	CPE	%CPE	Screened	CNSE	%CNSE	CPE	%CPE
Hospital-based laboratory										
*Escherichia coli*	2,560	15	0.6	3	0.12	2,537	26	1.0	1	0.04
*Klebsiella pneumoniae*	629	35	5.6	18	2.86	434	36	8.3	9	2.07
*Klebsiella oxytoca*	216	3	1.4	2	0.93	209	3	1.4	1	0.48
*Citrobacter* spp.	150	7	4.7	3	2.00	150	11	7.3	0	0
*Enterobacter* spp.	423	61	14.4	0	0	391	118	30.2	0	0
*Proteaceae*	551	7	1.3	0	0	559	15	2.7	0	0
Others	176	1	0.6	0	0	191	7	3.7	0	0
**Total**	**4,705**	**129**	**2.7**	**26**	**0.55**	**4,471**	**216**	**4.8**	**11**	**0.25**
Private community-serving laboratory^a^										
*Escherichia coli*	1,276	3	0.2	1	0.08	Not available				
*Klebsiella pneumoniae*	275	19	6.9	8	2.91					
*Klebsiella oxytoca*	73	3	4.1	1	1.37					
*Citrobacter* spp.	71	2	2.8	2	2.82					
*Enterobacter* spp.	81	10	12.3	0	0					
*Proteaceae*	184	0	0	0	0					
Others	31	0	0	0	0					
**Total**	**1,991**	**37**	**1.9**	**12**	**0.60**					

CNSE: carbapenem-non-susceptible *Enterobacteriaceae*; CPE, carbapenemase-producing *Enterobacteriaceae*; EUCAST: European Committee on Antimicrobial Susceptibility Testing.

^a^ Private laboratories served general practitioners for outpatient care.

The global proportion of CNSE among clinical *Enterobacteriaceae* isolates among hospitalised patients in 2015 was 2.7% (129/4,705; 95% confidence interval (CI): 2.3–3.2%) and it ranged per centre from 0.5% (1/200) to 7.0% (14/200). Compared with the 2012 results calculated after using identical microbiological criteria (216/4,471; 4.8%; 95% CI: 4.2–5.4%), the overall proportion of clinical CNSE decreased significantly in the same hospitals (p < 0.001 by Pearson chi-squared test). While the proportion of *Enterobacter* spp. among screened isolates in hospitals were identical between the two study periods (i.e. 9%; 423/4,705 in 2015 and 391/4,471 in 2012), the carbapenem non-susceptibility rate dropped significantly in this group from 30.2% (118/391) in 2012 to 14.4% (61/423) in 2015 (p < 0.001) and contributed to the overall decrease of the CNSE rate in 2015. 

Among ambulant patients, a total of 37 isolates from seven private laboratories would be categorised as CNSE representing a proportion of clinical CNSE isolates of 1.9% (37/1,991; 95% CI: 1.3%–2.5%) in the ambulatory setting ranging per centre from 0% to 8% (16/200). Comparing to the hospitals in which *Enterobacter* spp. accounted for nearly half of the CNSE isolates (61/129), *K. pneumoniae* represented the main CNSE species among the ambulatory isolates (19/37) since a lower proportion of *Enterobacter* spp. (4%; 81/1,991) were cultured in private laboratories. In hospitalised patients, the rate of meropenem non-susceptibility found in *K. pneumoniae* (2.5%; 16/629) remained stable in 2015 compared with 2012 (2.3%; 10/434).

Overall in 2015, 114/133 (86%) and 30/42 (71%) PCPE (using EUCAST screening cut-offs) isolates were referred from hospital-based and from private laboratories respectively to the reference laboratory and tested for the presence of carbapenemase. Considering the hospital-based and private laboratory data together, *K. pneumoniae* (26/38; 68%) and OXA-48 (28/38; 74%) were the main species and carbapenemase type respectively detected among CPE isolates in Belgium. While none of the 57 *Enterobacter* spp. isolates analysed could be confirmed as CPE, nearly half of the referred *K. pneumoniae* isolates (26/54; 48%) were confirmed as CPE (OXA-48-like, n = 17; KPC-type, n = 7; and NDM-type, n = 2). OXA-48 carbapenemase was detected in four *E. coli* isolates including one coproducing NDM enzyme, three *Citrobacter freundii*, two *C. koseri* and two *K. oxytoca* isolates. One VIM-producing *K. oxytoca* was also detected by PCR. All PCR-negative PCPE isolates did not hydrolyse imipenem by BYG Carba test, thus excluding the phenotypical expression of undetected carbapenemases by our multiplex PCR assay.

On the whole, the minimal estimated proportion of CPE was 0.55% (26/4,705; 95% CI: 0.34–0.76%) among clinical *Enterobacteriaceae* isolated from hospitalised patients and 0.60% (12/1,991; 95% CI: 0.26–0.94%) among the ambulatory patients ranging per centre from 0% (for 11 hospitals and seven private laboratories) to 3.09% (6/194; in one centre). The distribution of carbapenemase types by centre is listed in Table 2.

**Table 2:** Carbapenemase types detected in clinical CPE isolates per health centre, Belgium, 2012 (n = 11 CPE isolates) and 2015 (n = 38 CPE isolates)

Centre numbers	Carbapenemase (number of isolates)	
	2015 survey (38)	2012 survey (11)
Hospital-based laboratory		
C02	OXA-48 (1)	None
C04	None	KPC (1)
C06	OXA-48 (2)	OXA-48 (1)
C07	NDM-1 (1)	None
C08	NDM-5 and OXA-181 (1), OXA-48 (1)	OXA-48 (1)
C10	OXA-48 (1)	None
C11	OXA-48 (2)	None
C12	NDM-1 (1)	KPC (1)
C13	OXA-48 (2)	OXA-48 (1)
C14	KPC-2 (1), KPC-3 (1)	KPC (2), NDM (1)
C17	OXA-48 (4)	None
C19	None	OXA-48 (1)
C20	OXA-48 (1)	None
C23	KPC-3 (3), KPC-2 (1), VIM-1 (1)	OXA-48 (2)
C24	OXA-48 (2)	None
C01, C03, C05, C09, C15, C16, C18, C21, C22	None	None
Private community-serving laboratory^a^		
C26	OXA-48 (4)	Not available
C30	OXA-48 (6)	
C33	OXA-48 (1), KPC-3 (1)	
C25, C27, C28, C29, C31, C32, C34	None	

CPE: carbapenemase-producing *Enterobacteriaceae.*

^a^ Private laboratories served general practitioners for outpatient care.

In 2015 among hospitals, while the 16 cases of OXA-48 CPE were distributed in nine different hospitals, the 6 KPC-type CPE cases were grouped within two hospitals. In the community setting, the 12 CPE isolates (dominated by OXA-48; n = 11) were found in isolates from three private laboratories. 

Compared with 2012 (11/4,471, 0.25%; 95% CI: 0.10–0.39%), the overall proportion of clinical CPE in 2015 (26/4,705, 0.55%; 95% CI: 0.34–0.76%) increased significantly (p = 0.02 by Pearson chi-squared test) in the 24 surveyed hospitals and the proportion of hospitals collecting at least one clinical CPE isolate also appears to have increased from 8/24 in 2012 to 13/24 in 2015 (p value non-significant).

Based on disc diffusion with EUCAST interpretative criteria, all 38 confirmed CPE isolates in 2015 were intermediately-resistant or resistant to ertapenem disk while 16 (42%; all of OXA-48 type) were categorised as susceptible to meropenem disk. Two OXA-48-producing (one *E.* coli and one *C. koseri*) isolates had an inhibition zone to meropenem of ≥ 25 mm and would be missed if EUCAST screening breakpoint was applied using meropenem disc alone. One of these two CPE (*C. koseri*) showed additionally an ertapenem zone diameter of 24 mm and therefore would have been missed as well if the CLSI susceptibility breakpoints (ertapenem <22 mm and/or meropenem <23 mm) used as screening criteria in the 2012 survey were applied.

Origins and microbiological characteristics including MIC results of the 38 confirmed CPE isolates are detailed in Table 3.

**Table 3:** Samples data of confirmed CPE isolates and broth microdilution minimum inhibitory concentration (MIC) determination to 17 antimicrobials, Belgium, 2015 (n = 38)

Centre number	Sample origin	Hospital ward	Bacterial species	Carbapenemase	DiversiLab type^a^	Antimicrobial^b^ MICs (µg/mL)																
						TMC^c^	PTZ	CZT	CTX	CAZ	FEP	ATM	ETP	IPM	MEM	GEN	TOB	AMK	CIP	SXT	TGC	CST
C02	Urine	Medical	*K. pneumoniae*	OXA-48	5	> 1,024	256	32	> 32	64	64	128	4	4	2	> 8	16	4	> 16	> 4	0.25	0.5
C06	Respiratory	ICU	*K. pneumoniae*	OXA-48	3	> 1,024	> 256	16	> 32	16	16	64	4	4	2	> 8	16	4	4	> 4	0.25	0.5
C06	Urine	Medical	*K. pneumoniae*	OXA-48	Singleton	> 1,024	> 256	32	> 32	32	16	128	4	4	2	> 8	16	2	> 16	> 4	0.5	0.5
C07	Respiratory	ICU	*K. pneumoniae*	NDM-1	Singleton	48	> 256	> 64	> 32	> 64	64	32	> 4	32	32	1	8	8	1	0.5	0.5	0.5
C08	Respiratory	Medical	*E. coli*	NDM-5, OXA-181	NA	> 1,024	> 256	> 64	> 32	> 64	> 64	> 128	> 4	16	32	1	8	4	> 16	> 4	0.25	0.5
C08	Pus	Medical	*E. coli* ^d^	OXA-48	NA	> 1,024	64	0.25	1	0.5	0.5	1	1	1	0.5	> 8	4	2	> 16	1	0.25	4
C10	Urine	Medical	*C. freundii*	OXA-48	NA	> 1,024	> 256	16	> 32	16	> 64	64	1	2	1	1	8	4	1	> 4	0.25	0.5
C11	Other	Other	*K. pneumoniae*	OXA-48	Singleton	> 1,024	> 256	1	1	0.5	0.5	1	> 4	4	2	1	0.25	2	0.12	> 4	0.25	0.5
C11	Urine	Other	*C. freundii*	OXA-48	NA	> 1,024	> 256	2	4	2	1	1	2	4	2	> 8	4	2	8	> 4	2	0.5
C12	Pus	Medical	*K. pneumoniae*	NDM-1	Singleton	> 1,024	> 256	> 64	> 32	> 64	64	> 128	> 4	32	16	> 8	> 32	> 128	> 16	2	0.25	0.5
C13	Urine	ICU	*K. oxytoca*	OXA-48	NA	> 1,024	> 256	32	32	> 64	4	> 128	> 4	4	4	1	0.25	2	2	0.5	1	0.5
C13	Pus	Other	*K. pneumoniae*	OXA-48	Singleton	> 1,024	> 256	64	> 32	64	64	128	> 4	8	4	> 8	8	2	> 16	> 4	1	> 16
C14	Respiratory	ICU	*K. pneumoniae*	KPC-2	Singleton	96	> 256	64	> 32	> 64	> 64	> 128	> 4	64	> 32	2	1	4	> 16	1	1	8
C14	Other	ICU	*K. pneumoniae*	KPC-3	Singleton	48	> 256	> 64	> 32	> 64	32	> 128	> 4	16	8	> 8	32	4	4	> 4	0.25	0.5
C17	Urine	Medical	*K. pneumoniae*	OXA-48	23	> 1,024	256	1	1	0.5	0.5	1	2	4	1	1	0.25	2	16	> 4	0.5	0.5
C17	Urine	Medical	*K. pneumoniae*	OXA-48	23	> 1,024	> 256	32	> 32	64	64	128	2	2	2	1	4	2	> 16	> 4	1	0.5
C17	Pus	Surgery	*K. pneumoniae*	OXA-48	Singleton	> 1,024	> 256	32	> 32	64	32	> 128	4	4	2	1	8	2	> 16	0.5	0.5	0.5
C17	Urine	Medical	*K. pneumoniae*	OXA-48	23	> 1,024	> 256	16	> 32	16	64	64	1	4	2	1	8	2	> 16	0.5	0.5	0.5
C20	Respiratory	Medical	*K. pneumoniae*	OXA-48	Singleton	> 1,024	256	16	> 32	32	16	64	1	2	0.5	> 8	32	4	> 16	> 4	0.25	> 16
C23	Urine	Medical	*K. pneumoniae*	KPC-3	16	48	256	> 64	> 32	64	64	128	> 4	32	> 32	> 8	> 32	32	> 16	> 4	0.5	16
C23	Pus	ICU	*K. pneumoniae*	KPC-2	Singleton	24	256	16	16	16	16	128	> 4	8	8	1	0.5	2	0.25	0.5	2	0.5
C23	Pus	Medical	*K. pneumoniae*	KPC-3	16	32	256	64	> 32	64	64	128	> 4	32	> 32	> 8	32	32	> 16	> 4	1	16
C23	Respiratory	Medical	*K. pneumoniae*	KPC-3	16	32	256	64	> 32	64	64	128	> 4	32	> 32	> 8	32	64	> 16	> 4	1	> 16
C23	Respiratory	Medical	*K. oxytoca*	VIM-1	NA	256	256	> 64	> 32	> 64	16	128	0.5	8	2	1	8	8	0.5	> 4	0.25	0.5
C24	Blood	Other	*E. coli*	OXA-48	NA	> 1,024	128	1	1	0.5	0.5	1	2	0.5	1	1	0.25	2	> 16	> 4	0.25	0.5
C24	Other	Other	*C. koseri*	OXA-48	NA	> 1,024	256	1	4	1	2	4	> 4	1	1	1	1	4	0.25	> 4	0.25	1
C26	Urine	Ambulatory	*K. pneumoniae*	OXA-48	Singleton	768	> 256	2	1	0.5	0.5	1	2	8	4	1	0.5	2	0.12	0.5	0.25	1
C26	Urine	Ambulatory	*K. oxytoca*	OXA-48	NA	> 1,024	256	4	2	2	2	64	1	1	0.5	1	0.25	2	4	> 4	0.5	0.5
C26	Urine	Ambulatory	*K. pneumoniae*	OXA-48	5	> 1,024	128	16	> 32	32	16	64	1	0.5	0.25	> 8	> 32	16	> 16	> 4	0.25	0.5
C26	Urine	Ambulatory	*K. pneumoniae*	OXA-48	5	> 1,024	256	32	> 32	32	64	64	4	4	4	> 8	32	32	> 16	> 4	0.5	0.5
C30	Urine	Ambulatory	*K. pneumoniae*	OXA-48	Singleton	> 1,024	128	1	1	0.5	0.5	1	1	0.5	0.25	1	16	4	16	0.5	0.25	0.5
C30	Urine	Ambulatory	*C. koseri*	OXA-48	NA	256	16	0.25	1	0.5	0.5	1	0.25	0.5	0.25	1	0.25	2	0.12	0.5	0.25	0.5
C30	Pus	Ambulatory	*K. pneumoniae*	OXA-48	3	> 1,024	128	16	> 32	32	32	64	2	0.5	0.5	> 8	32	4	> 16	> 4	0.5	0.5
C30	Urine	Ambulatory	*K. pneumoniae*	OXA-48	3	> 1,024	> 256	16	> 32	16	16	32	2	2	1	> 8	16	4	4	> 4	0.5	0.5
C30	Pus	Ambulatory	*K. pneumoniae*	OXA-48	3	> 1,024	128	2	32	4	4	32	1	2	0.5	> 8	16	2	2	> 4	0.25	0.5
C30	Urine	Ambulatory	*C. freundii*	OXA-48	NA	> 1,024	256	4	16	32	0.5	32	2	4	2	4	16	32	1	0.5	0.25	0.5
C33	Urine	Ambulatory	*E. coli*	OXA-48	NA	512	32	0.25	1	0.5	0.5	1	1	0.5	0.25	1	0.5	2	0.12	0.5	0.25	0.5
C33	Urine	Ambulatory	*K. pneumoniae*	KPC-3	Singleton	32	256	32	8	64	4	128	> 4	8	8	1	32	32	> 16	> 4	0.5	0.5

*C. freundii: Citrobacter freundii; C. koseri: Citrobacter koseri;* CPE: carbapenemase-producing *Enterobacteriaceae; E. coli*: *Escherichia coli*; ICU (intensive care unit); *K. pneumoniae*: *Klebsiella pneumoniae;K. oxytoca*: *Klebsiella oxytoca*; NA: not available.

^a^ Singleton, type harboured by a single strain.

^b^ AMK: amikacin; AZT: aztreonam; CAZ: ceftazidime; CIP: ciprofloxacin; CST: colistin; CTX: cefotaxime; CZT: ceftolozane/tazobactam; ETP: ertapenem; FEP: cefepime; GEN: gentamicin; IPM: imipenem; MEM: meropenem; SXT: trimethoprim/sulfamethoxazole; TGC: tigecycline; TMC: temocillin; TOB: tobramycin; TZP: piperacillin/tazobactam.

^c^ MIC determined by Etest method for temocillin.

^d^ Colistin-resistant MCR-1-positive strain.

On the basis of MIC using EUCAST interpretative criteria, only two (5%; one VIM-producing *K. oxytoca* and one OXA-48-producing *C. koseri*) isolates were susceptible to ertapenem (MIC ≤1 µg/mL), while 23 (61%) OXA-48-positive isolates had imipenem and/or meropenem MICs within the susceptible range (MIC ≤2 µg/mL). All CPE isolates were found resistant to piperacillin/tazobactam and to temocillin with higher temocillin MIC levels (≥ 256 µg/mL) for OXA-48 and VIM producers compared with more variable MIC levels (24–1,024 µg/mL) for KPC- and for NDM-positive isolates. Ceftolozane/tazobactam showed poor activity against CPE since only eight (21%) OXA-48 producers had MIC below the susceptibility cut-off (≤ 1 µg/mL). Tigecycline retained the highest in vitro activity (MIC ≤1 µg/mL) against 95% (36/38) of CPE followed by colistin (MIC ≤2 µg/mL), which was active against 82% (31/38) of the CPE. Interestingly of the seven colistin-resistant CPE isolates in 2015, which were all collected from hospitals (only one detected in the 2012 survey), one OXA-48-positive *E. coli* was found positive for the plasmid-mediated *mcr-1* gene.

Molecular typing by rep-PCR of the 26 carbapenemase-producing *K. pneumoniae* isolates showed their belonging to 19 different DiversiLab (DL) patterns with four clusters (DL types 3, 5, 16 and 23) of more than one isolates (Table 3). The three DL type 16 KPC-3 producing isolates were detected in centre C23 and the three DL type 23 OXA-48 producing isolates in centre C17. The other DL types 3 and 5 (all OXA-48 producing) isolates were found in two (one hospital-based and one community-serving) centres each.

## Discussion

In this multicentric 2015 survey, we found an overall doubling proportion of 0.55% of clinical CPE among *Enterobacteriaceae* isolates in hospitals compared with 2012 (0.25%). The microbiological analysis confirmed the predominance of *K. pneumoniae* and of OXA-48 among CPE isolates in hospitals and in the community in Belgium. Molecular typing of carbapenemase-producing *K. pneumoniae* confirmed mostly polyclonal distribution although clusters of identical DL types with suspected clonal spread were highlighted as well. Two clusters of different DL types (16 and 23) strongly suggested their intra-hospital clonal dissemination or outbreak. Additionally, the presence of other OXA-48 producing DL types (3 and 5) both in hospitals and in private laboratories that are geographically close (distance < 30 km) raised the possibility of epidemiological links between the two healthcare sectors. Although no patient history regarding travel or hospitalisation in foreign country was available in this study, data from the national surveillance programme and from the previous prevalence study in 2012 support that the proportion of travel-imported CPE cases should be limited [17].

The strength of our study relies on the use of a standardised methodology performed within the same hospitals between the two study periods to measure the epidemiological parameters with minimal variations due to technical inter-laboratory variability or samples selection bias. The 24 hospital-based laboratories serving tertiary care and general acute-care hospitals of medium to large size were selected for their representative distribution in the three geographical regions (Flanders, Brussels and Wallonia) across Belgium. Only strains isolated from clinical samples were included in the studies in order to limit recruitment bias due to variations in screening strategies for CPE between hospitals. However, our study has some limitations. First, 18% (31/175) of the PCPE isolates detected locally were not referred to the reference laboratory suggesting that the proportion of CPE of 0.55% should be considered as a minimal estimate. Also, the higher proportion of CPE in hospitals in 2015 compared to 2012 could result from the use of EUCAST screening breakpoints theoretically more sensitive than the CLSI susceptibility criteria applied in 2012. In spite of this, the fact that in the present study, only one OXA-48 producing *C. koseri* had the two carbapenems inhibition zones above the CLSI susceptibility cut-offs suggests very rare occurrence of these CPE isolates that poorly expressed carbapenemases.

In addition, the higher proportion of colistin resistance among CPE strains detected in hospitals corroborates with the phenomenon of increasing colistin resistance among CPE isolates observed in Belgium and elsewhere for the past years [18,19]. Furthermore the study evidenced the emergence of the first human MCR-1-positive CPE in Belgian hospitals as well, similar other countries worldwide, where this has also been recently reported in healthcare [20-22].

In Spain, the latest prospective multicentric study in 83 hospitals performed in 2013 reported a similar overall estimated prevalence of 0.3% of CPE [4] (higher than the 0.04% of metallo-beta-lactamase-producing CPE detected in the previous 35-centres survey in 2009) [23], but 20% of the CPE were of faecal screening origin. The survey showed the same predominance of OXA-48 type CPE, but VIM-producing isolates (the second most frequent) were more widely geographically distributed. Sporadic community-onset infection and post-travel acquisition of CPE had been reported in western countries [24-26]. In 2016 a case report in Belgium described a household acquisition of OXA-48 producing *K. pneumoniae* in a woman living in the same household than a patient previously infected with a similar CPE, raising the possibility of community spread in low-prevalence areas [27]. However to our knowledge, no specific prospective cross-sectional survey addressing clinical isolates in an ambulatory setting has yet been published.

Regarding the smaller proportion of CNSE (mainly due to the significant decreasing proportion of CNSE among *Enterobacter* spp.) in hospitals in 2015 compared with 2012, no clear explanation could be found. However, a cluster effect could be excluded since the decreased rate of CNSE was documented in 18 of the 24 participating hospitals (data not shown).

In conclusion, this multicentric survey demonstrated a significantly increased proportion of CPE among clinical *Enterobacteriaceae* isolates in hospitals from 2012 to 2015 with an increased proportion of hospitals-based laboratories detecting at least one CPE. Although the proportion of CPE remained globally low, the steady progression and spread of CPE in Belgian hospitals and the emergence of colistin-resistant (including plasmid-mediated *mcr-1* harbouring) strains among CPE raises major concerns. In parallel, the survey in 10 private laboratories highlighted for the first time the establishment of CPE (mainly *K. pneumoniae* species and of OXA-48 type) in an ambulatory setting and suggested possible epidemiological links with hospitals. We therefore believe that repeated national or regional epidemiological studies using standardised protocols at different healthcare levels are essential to measure more accurately the burden of carbapenem resistance.

## Notes

**Multicentre Study Group:** An Boel and Kristien Van Vaerenbergh (OLV Aalst); Margareta Ieven and Herman Goossens (UZ Antwerpen); Jean-Sébastien Goffinet (CSL Arlon); Johan Frans (Imeldaziekenhuis Bonheiden); Yves De Gheldre (CHIREC, Braine-L’Alleud); Eric Nulens (AZ St-Jan, Brugge); Yvette Miendje, Massin Lebitasy and Georges Mascart (CHU Brugmann/HUDERF, Brussels); Helga Kandet-Yattara and Olivier Vandenberg (CHU St-Pierre, Brussels); Hector Rodriguez-Villalobos and Anne Simon (CU St-Luc UCL, Brussels); Claire Nonhoff and Olivier Denis (Hôpital Erasme, Brussels); Deborah de Geyter and Denis Pierard (UZ Brussel, Brussels); B. Lissoir (GHDC Charleroi); Niels Graindor and Guy Coppens (ZOL Genk); Anne-Marie Vandenabeele (AZ St-Lucas, Gent); Jerina Boelens and Geert Claeys (UZ Gent); Koen Magerman (Jessa Ziekenhuis Hasselt); Anne Pernet and Catherine Potvliege (CHU Tivoli, La Louviere); Veroniek Saegeman and Jan Verhaegen (UZ Leuven); Pierre Gavage and Jean-Marc Senterre (CHR Citadelle, Liege); Cecile Meex and Pierrette Melin (CHU Sart-Tilman, Liege); Maria-Grazia Garrino (CHR Namur, Namur); Terry Laurent (CMSE, Namur); Valerie Verbelen (Cliniques St-Luc, Ottignies); Te-Din Huang and Youri Glupczynski (CHU Dinant-Godinne, Yvoir); Brigitte Claude (IBC, Brussels); Colette Jouniaux (CPG, Brussels); Evilien Vekens (Medina Dendermonde); Herman Nuytten (Nuytinck, Evergem); Ludo Mertens (KLaboRigo, Genk); Martine Thys (CMA Herentals); Annemie Vandermeersch and Gudrun Depourcq (Bruyland, Kortrijk); Linda Joosten (AKL, Lier); Sophie Woestyn (Laboratoire J. Woestyn, Mouscron); Dominique Cuigniez (Medilab, Sint-Niklaas).
